# Impact of Preoperative Radiological Assessment on Surgical Outcomes in Orthopedic Procedures: A Retrospective Study

**DOI:** 10.7759/cureus.96283

**Published:** 2025-11-07

**Authors:** Syed Taqi Askari Shah, Kaleem Ullah, Muhammad Zeeshan Akram, Syed Hassan Raza Bokhari, Muhammad Umer Khan Khalil, Abdul Samad Qureshi, Tayyaba Fazal, Taimoor Ahmad Rana, Sultan Mehmood

**Affiliations:** 1 Trauma and Orthopaedics, Queen's Medical Centre, Nottingham, GBR; 2 Orthopaedic Surgery, Gujranwala Teaching Hospital, Gujranwala, PAK; 3 Trauma and Orthopaedics, King Edward Medical University, Lahore, PAK; 4 Trauma and Orthopaedics, East Kent University Hospitals, Kent, GBR; 5 Radiology, Northwest School of Medicine, Peshawar, PAK; 6 Orthopaedics, Indus Medical College, Tando Muhammad Khan, PAK; 7 Radiology, Shaheed Zulfiqar Ali Bhutto Medical University, Islamabad, PAK; 8 Internal Medicine, Aziz Bhatti Shaheed Teaching Hospital, Gujrat, PAK; 9 Internal Medicine, Rural Health Center Malka, Kharian, PAK

**Keywords:** dexa, orthopedic, preoperative, radiology, surgical outcomes

## Abstract

Background: Preoperative radiological assessment plays a crucial role in orthopedic surgery by providing detailed anatomical insights and guiding surgical planning.

Objective: This study aimed to assess the impact of preoperative radiological assessment on surgical outcomes in patients undergoing orthopedic procedures.

Methods: This retrospective study was conducted at Shaheed Zulfiqar Ali Bhutto Medical University from June 2024 to December 2024, including 143 patients. Imaging quality was classified as adequate or inadequate based on completeness, correct projections, diagnostic clarity, and alignment with surgical objectives. Data extracted from electronic medical records included demographic characteristics, intraoperative parameters (duration of surgery, blood loss), and postoperative outcomes (infection, implant alignment, length of stay, functional recovery). Group comparisons were made using appropriate statistical tests, and 95% confidence intervals (CIs) were calculated for the main outcomes.

Results: Out of the 143 patients, 98 (68.5%) had adequate imaging, and 45 (31.5%) had inadequate imaging. The adequately imaged group showed shorter operative time (88.6±17.5 vs. 106.2±21.3 minutes; p<0.001; 95% CI: −25.7 to −9.5) and less blood loss (285.4±64.3 vs. 354.1±77.2 mL; p=0.004; 95% CI: −112.3 to −34.7). Postoperative wound infections occurred in 5/98 (5.1%) vs. 7/45 (15.5%) (p=0.041; 95% CI for difference: −20% to −1.2%), and implant misalignment in 3/98 (3.1%) vs. 6/45 (13.3%) (p=0.026). The adequately imaged group also had a shorter mean hospital stay (4.1±1.3 vs. 5.3±1.6 days; p=0.002) and higher functional recovery scores (84.6±7.2 vs. 77.9±9.1; p=0.009).

Conclusion: Adequate preoperative radiological assessment is associated with improved surgical efficiency, reduced complications, and improved postoperative outcomes in orthopedic procedures. These findings support the routine implementation of structured imaging protocols as a standard of care in orthopedic surgical planning.

## Introduction

The work of preoperative planning in the context of contemporary orthopedic surgery cannot be overestimated, particularly when it deals with further increasing the accuracy of surgical procedures and reducing the number of complications [1]. Radiological assessment is one of the major parts of this planning; it forms the foundation around which the anatomical area will be measured and possible expectations of both difficulties during the surgery and after the procedure are determined [2,3]. Whether it is as basic as simple fracture fixation or as complex as joint replacements and spinal surgeries, radiological imaging is invaluable in affording one a window into the structural integrity, alignment, and bone density of the musculoskeletal system and the pathological changes occurring to the system [4]. Recent improvements in imaging modalities like digital radiography, computed tomography (CT), magnetic resonance imaging (MRI), dual-energy X-ray absorptiometry (DEXA), and intraoperative fluoroscopy have changed orthopedic diagnostics [5]. Not only do these devices enhance diagnostic quality, but they also help implement patient-specific surgical planning based on the anatomy of an individual patient and the individual's pathology. As an example, CT scans before the operation in acetabular fractures are beneficial in defining patterns of the fracture and the best fixation method, whereas MRI is quite useful in evaluating the degree of involvement of soft tissues in rotator cuff tears, meniscal injuries, and spinal cord disorders [6]. Such depth of visualization enables orthopedic surgeons to pre-determine the operative approach, proper instrumentation, and cases that are potentially high risk [7].

Other studies that have produced high impacts reported that a thorough preoperative imaging has significantly improved the decision-making intraoperatively and resulted in desirable clinical outcomes [8]. As an example, a proper radiographic templating prior to total hip arthroplasty (THA) may assist in optimal planning of the actual size and alignment of prosthesis components, thus avoiding the chances of leg length discrepancies, impingement, or premature failure of the prostheses [9]. In the setting of spinal surgeries, especially corrective and instrumentation of deformity, preoperative MRI and CT help to assess the compromise of the spinal canal, nerve root compression, and the anatomy of the vertebra and have a direct impact on the safety and effectiveness of the operation. In addition to this, radiological preoperative assessment is not only essential in elective surgery but is progressively essential in trauma [10]. Detailed imaging plays a vital role in distinguishing between polytrauma and complex fractures, enabling the prioritization of surgical procedures, detection of occult injuries, and assessment of bony stability. It helps reduce surgical duration, minimize intraoperative surprises, and improve hemodynamic control in unstable patients [11,12]. Fracture radiographic classification, like the Arbeitsgemeinschaft für Osteosynthesefragen (AO)/Orthopaedic Trauma Association (OTA) Fracture and Dislocation Classification System, and osteoarthritis grading, as per Kellgren and Lawrence, are completely dependent on radiographic imaging and further display how standardized radiology helps in clinical decision-making [13]. Nevertheless, the implementation of radiology planning is not always consistent since it is frequently not used in clinical practice because it can be costly and time-consuming and is not always available to radiologists or the radiologist may not be trained to use it. In low- and middle-income countries (LMICs), in particular, orthopedic workload is high, and radiographic coverage may be poor, so many procedures are carried out on the basis of constrained radiographic information. It results in the higher risks of malalignment, wrong positioning of an implant, non-union, or revision surgery, all of which cause healthcare spending and patient morbidity [14,15].

Objective

The main objective of the study is to assess the impact of preoperative radiological assessment on surgical outcomes in patients undergoing orthopedic procedures.

## Materials and methods

This was a retrospective study conducted at Shaheed Zulfiqar Ali Bhutto Medical University, Islamabad, Pakistan, from June 2024 to December 2024 after obtaining approval from the institute's Ethics Review Board (approval number: F. 1-1/2022/ERB/SZABMU/715). A total of 143 patients undergoing orthopedic surgical procedures were included in this study. The sample size was calculated using the World Health Organization (WHO) sample size calculator. With a 95% confidence level, a 5% margin of error, and an anticipated complication rate of 20%, the minimum required sample size was 138. A total of 143 patients were included to account for potential data exclusions and ensure adequate power. Patient records were identified through the hospital's electronic medical record (EMR) system and operative logs using procedure-specific Current Procedural Terminology (CPT) codes and diagnostic keywords such as "fracture fixation", "arthroplasty", and "spinal instrumentation". Consecutive cases meeting the inclusion criteria during the study period were reviewed. Duplicates and incomplete entries were excluded. Non-probability consecutive sampling was employed to enroll all eligible patients (Table 1).

**Table 1:** Inclusion and exclusion criteria for the study participants

Inclusion criteria	Exclusion criteria
Patients of either gender, aged 18 years and above	Incomplete or missing radiological records
Patients who underwent orthopedic surgery (trauma, joint replacement, spinal surgery)	Emergency surgical cases with no prior imaging
Availability of complete preoperative radiological records	Patients with pathological fractures due to malignancy or infection
Availability of detailed postoperative follow-up data (≥30 days)	Revision surgeries or reoperations

Data collection procedure

Patient data were extracted from the hospital's EMRs and operative documentation. Key variables collected included demographic details, type and quality of preoperative imaging (X-ray, CT scan, MRI), intraoperative events such as duration of surgery and blood loss, any immediate surgical complications, and postoperative outcomes including infection, implant position, length of hospital stay, and functional recovery scores. Collected variables included demographics (age, gender); comorbidities such as diabetes mellitus, hypertension, obesity (BMI ≥30 kg/m²), and smoking status; and surgeon-related factors, including years of independent surgical experience categorized as ≤5 years or >5 years. The radiological assessments were evaluated for completeness and clarity and were then classified into two categories, namely, "adequate" and "inadequate", based on criteria such as correct imaging angles, diagnostic resolution, and alignment with surgical objectives. The adequacy of each imaging record was independently reviewed by one consultant musculoskeletal radiologist and two orthopedic surgeons. To ensure objectivity, reviewers were blinded to intraoperative findings and postoperative outcomes during assessment. In cases of disagreement, a consensus was reached through a joint review session. Inter-observer reliability was measured using Cohen's kappa coefficient, yielding κ=0.82, indicating substantial agreement among raters and ensuring the reproducibility of the classification process.

Data analysis

All data were entered into IBM SPSS Statistics for Windows, Version 26.0 (Released 2019; IBM Corp., Armonk, New York, United States), for statistical analysis. Descriptive statistics such as mean, standard deviation, and frequency distributions were used to summarize baseline characteristics. To compare surgical outcomes between the adequate and inadequate radiology groups, independent t-tests or Mann-Whitney U tests were used for continuous variables, while chi-squared tests were applied to categorical data. A p-value of less than 0.05 was considered statistically significant. Subgroup analysis was also conducted based on procedure type and imaging modality.

## Results

Among 143 patients, both groups were demographically comparable in terms of age and gender (p>0.05). However, operative time was significantly longer in those with inadequate imaging (106.2 vs. 88.6 minutes; p<0.001), and they experienced greater blood loss (354.1 mL vs. 285.4 mL; p=0.004). Intraoperative complications were significantly more frequent in the inadequate imaging group (24.4% vs. 9.2%; p=0.018), suggesting that imaging quality directly influenced surgical safety (Table 2).

**Table 2:** Demographic and clinical characteristics of the patients (n=143) Values are presented as mean±standard deviation (SD) or number (percentage). The independent t-test was used to compare the means of continuous variables (age, operative time, blood loss) between the two groups. The chi-squared test was used to compare the proportions of categorical variables (gender, procedure type, intraoperative complications) between the two groups.

Characteristic	Adequate imaging (n=98)	Inadequate imaging (n=45)
Age (years), mean±SD	47.2±14.7	46.0±16.3
Gender (male), n (%)	59 (60.2%)	25 (55.6%)
Common procedure type, n (%)		
Fracture fixation	38 (38.8%)	21 (46.7%)
Joint arthroplasty	31 (31.6%)	9 (20%)
Spinal surgery	19 (19.4%)	6 (13.3%)
Operative time (min), mean±SD	88.6±17.5	106.2±21.3
Blood loss (mL), mean±SD	285.4±64.3	354.1±77.2
Intraoperative complications, n (%)	9 (9.2%)	11 (24.4%)

Wound infections occurred in seven (15.5%) of these patients compared to five (5.1%) in the adequately imaged group (p=0.041), while implant misalignment was also significantly higher (13.3% vs. 3.1%; p=0.026). Additionally, those with inadequate imaging had longer hospital stays (5.3 vs. 4.1 days; p=0.002) and lower functional recovery scores (77.9 vs. 84.6; p=0.009) (Table 3).

**Table 3:** Postoperative outcomes and functional results The independent t-test was applied for continuous variables (length of stay, functional score). The chi-squared (χ²) test was used for categorical variables (wound infection, implant malposition/misalignment). A p-value of <0.05 was considered statistically significant.

Outcome variable	Adequate imaging (n=98)	Inadequate imaging (n=45)	Test statistic	P-value
Wound infection, n (%)	5 (5.1%)	7 (15.5%)	χ²=4.15	0.041
Implant malposition/misalignment, n (%)	3 (3.1%)	6 (13.3%)	χ²=5.00	0.026
Length of stay (days), mean±SD	4.1±1.3	5.3±1.6	t=3.17	0.002
Functional score, mean±SD	84.6±7.2	77.9±9.1	t=2.65	0.009

Different procedures showed varying reliance on imaging modalities. X-ray was used in nearly all joint arthroplasty cases 40 (100%) and the majority of fracture fixation procedures (42, 71.2%). CT was used in about half the cases for both arthroplasty and fracture fixation. In contrast, MRI was predominantly utilized for spinal surgeries (19, 76%), which aligns with its superior soft tissue resolution (Table 4).

**Table 4:** Radiological modality used by procedure type (n=143) Values are expressed as number (%) of cases using each imaging modality. CT: computed tomography; MRI: magnetic resonance imaging

Procedure type	X-ray only n (%)	CT scan used n (%)	MRI used n (%)	Combined imaging n (%)
Fracture fixation (n=59)	42 (71.2%)	30 (50.8%)	2 (3.4%)	13 (22%)
Joint arthroplasty (n=40)	40 (100%)	18 (45%)	4 (10%)	16 (40%)
Spinal surgery (n=25)	5 (20%)	10 (40%)	19 (76%)	9 (36%)
Other procedures (n=19)	15 (78.9%)	4 (21%)	1 (5.2%)	2 (10.5%)

There was a strong and statistically significant relationship between imaging quality and complication rates (p=0.008). Patients with inadequate imaging experienced postoperative complications in 16 (35.6%) cases compared to only 13 (13.3%) in those with adequate imaging. This nearly threefold increase in risk, supported by a chi-squared value of 6.98, highlights the predictive value of imaging adequacy (Table 5).

**Table 5:** Association between imaging quality and postoperative complications (n=143) Values are presented as number (%) of patients within each imaging quality group. Comparison between adequate and inadequate imaging groups was performed using the chi-squared (χ²) test. A p-value of <0.05 was considered statistically significant.

Imaging quality	Any post-op complication n (%)	No complication n (%)	Total n (%)	Test statistic	P-value
Adequate (n=98)	13 (13.3%)	85 (86.7%)	98 (100%)	χ²=6.98	0.008
Inadequate (n=45)	16 (35.6%)	29 (64.4%)	45 (100%)		
Total	29 (20.3%)	114 (79.7%)	143 (100%)		

## Discussion

It was on this basis that a study was conducted to determine how radiological assessment before a surgical procedure affects other primary surgical outcomes in orthopedic operations, taking a sample of 143 patients. Our findings suggest an association between imaging adequacy and surgical outcomes: that quality and full imaging before an operation are the significant factors that enhance the work of the operation, decrease the risks of a complication, and enhance post-surgery recovery. Individuals who obtained satisfactory preoperative imaging performed better intraoperatively in terms of shorter duration of operation as well as reduced estimated blood loss. The average surgery length in the adequate group was found to be around 18 minutes less than that of the inadequate group, with a significant gap in mean analysis (p<0.001). This implies that higher levels of fidelity in the visualization of the anatomical structures and pathology will warrant the surgeons to perform the surgical procedures more effectively and with more confidence, decreasing unnecessary waiting time. The same patterns are described in the past studies, in which preoperative templating and 3D planning resulted in increased accuracy and decreased operating time in joint arthroplasty and spinal instrumentation [16,17].

Besides the intraoperative outcomes, the number of postoperative complications was significantly reduced in the group in which the radiological assessment was done completely. Considerably, there was a marked decrease in the levels of wound infection and malalignment of the implants in this group (p=0.041 and p=0.026, respectively). These findings are consistent with the findings of past studies that have highlighted the effects of incomplete or misinterpreted imaging on poor implant positioning, which may result in early mechanical failure or dismal functional outcomes or necessitate revision surgery [18]. Also, the mean hospital stay duration was considerably less in the adequately imaged group (4.1 days versus 5.3 days), thus further demonstrating the practical advantages of comprehensive imaging in postoperative recovery and hospital cost limitation. Patients who were subjected to sufficient imaging also had better functional outcomes as evaluated based on scoring systems of the procedures done (p=0.009). This reinforces the clinical understanding that precise surgical planning, particularly when guided by CT scans, MRI, or a combination of imaging modalities, leads to improved long-term outcomes and enhanced patient mobility. As an example, MRI assessment of the integrity of soft tissues prior to a shoulder or knee surgery has been demonstrated in the past literature to directly influence both the surgical strategy and the postoperative rehabilitation outcome. The most noticeable one was in Table 5, where the complication rate was much stronger between patients with inadequate imaging (35.6%) and adequate imaging (13.3%) (p=0.008). This directly contributes to the suggestion that bad imaging, or the absence of it, is not mere administrative sloppiness but is indeed a real differential from the clinical risk side. Earlier research has also come to the same findings, stating that poor imaging is associated with higher surgical complications, revision of implants, and medicolegal liabilities [19].

This study has several limitations that must be acknowledged when interpreting its findings. First, the retrospective design inherently limits the ability to establish causality between imaging adequacy and surgical outcomes. Because data were extracted from existing medical records, the accuracy of variables such as blood loss or operative time depended on documentation quality and may be prone to minor recording bias. Moreover, the adequacy of imaging was not only subjective but also measured using a checklist that was predefined but at the same time subjective, which might cause variation. However, the research provides evidence supporting the idea that the proper and regular, high-quality radiological examination must be regarded as a basic requirement preconditioning orthopedic surgery, particularly when complex or elective surgery is to be done.

## Conclusions

Adequate preoperative radiological assessment significantly improves surgical outcomes in orthopedic procedures. Patients who underwent comprehensive imaging experienced shorter operative times, reduced intraoperative blood loss, fewer complications, and better functional recovery compared to those with inadequate imaging. The study highlights the critical role of appropriate imaging modalities, particularly CT and MRI, in enhancing surgical precision, planning, and postoperative care.
